# Supplementary material for: Longitudinal evidence that Event Related Potential measures of self-regulation do not predict everyday goal pursuit
Source: Nat Commun. 2022 Jun 9;13:3201. doi: 10.1038/s41467-022-30786-7 (PMC9184581; doi:10.1038/s41467-022-30786-7)
Supplement: Supplementary file 1 — Supplementary Information [file 41467_2022_30786_MOESM1_ESM.pdf]

# Supplementary Information

**Saunders, Milyavskaya, & Inzlicht**

1. **Classic ERP and Flanker effects**
2. **Predicting goal progress from desire and resistance**
3. **Analyses with conflicting desires only**
4. **Alternate operationalizations of ERPs (i.e., ERN, RewP, LPP).**

## 1. Classic ERP and Flanker effects

We examined the behavioural and ERP data to ensure that our manipulations produced the canonical within-subjects effects (e.g., flanker effects on error rates and reaction times, error-related ERP effects). Participants were slower on incompatible (*M* = 488 ms, *S.D.* = 44 ms) compared to compatible flanker trials (*M* = 401 ms, *S.D.* = 34 ms), $t(180) = -47.30$, $p < .001$, $d = -3.52$, and made more errors on incompatible (*M* = 18.8%, *S.D.* = 12.6%) than compatible trials (*M* = 2.1%, *S.D.* = 3.4%), $t(180) = -20.09$, $p < .001$, $d = -1.49$. The ERN was significantly more negative on error (*M* = -7.31 µV, *S.D.* = 5.74) than correct trials (*M* = 3.06 µV, S.D. = 3.66), $t(169) = 20.42$, $p < .001$, $d = 1.57$, see Figure 1, upper panel.

The two stimulus-locked ERPs (RewP and LPP also demonstrated the expected effects. The RewP was also more positive to correct feedback (*M* = 11.03 µV, *S.D.* = 7.96) than to error feedback (*M* = 5.67 µV, *S.D.* = 6.62) on the time-estimation task, $t(188) = 16.73$, $p < .001$, $d = 1.22$, see Figure 1, lower panel. A 2 (valence: positive vs. negative) x 2 (arousal: high vs. low) x 2 (window: early LPP vs. late LPP) repeated-measures ANOVA was used to assess the LPP across image types revealed a significant three-way interaction, $F(1, 189) = 23.34$, $p < .001$. As observed in Figure 2, the LPP was larger in amplitude in the early compared to late window, and was considerably larger for high arousal than low arousal affective stimuli.

## 2. Predicting goal progress from desire and resistance

Following our preregistration, in an attempt to conceptually replicate our prior work (Milyavskaya & Inzlicht, 2017), we conducted a series of regressions with desire frequency/strength and resistance strength entered as predictors of goal progress. None of the regressions were significant (see OSF for full output). This is likely because in this study we examined all desires, irrespective of conflict with goals, whereas Milyavskaya & Inzlicht (2017) used desires that conflicted with personal goals (as a measure of temptation). In an effort to better understand these findings, and to allow for closer comparison with previous work, we also computed a variable for temptation in the same way as Milyavskaya & Inzlicht (2017), by multiplying average conflict with desire. Regression analyses with temptation and resistance predicting goal progress replicated Milyavskaya & Inzlicht, 2017, showing that temptation, but not resistance, was consistently related to goal progress, $\beta$s = -.26 to -.28 (see Table S1 below). That is, people who experienced many conflicting desires, many temptations, were worse at reaching their goals; whether they tried to restrain their desires (or not) did not make a difference. Replicating past work, in-the-moment self-control made little difference in goal progress (Milyavskaya & Inzlicht, 2017).

Table S1: Predicting goal progress from desire and resistance

| | goal progress at 1 month | | | | | goal progress at 3 months | | | | | goal progress at 6 months | | | | |
|---|---|---|---|---|---|---|---|---|---|---|---|---|---|---|---|
| | B | 95%CI | beta | t | p | B | 95%CI | beta | t | p | B | 95%CI | beta | t | p |
| Average resistance | -.05 | [-.21;11] | -.06 | -0.64 | .522 | -.03 | [-.21;.14] | -.03 | -.35 | .725 | -.09 | [-.31;.13] | -.08 | -.84 | .403 |
| Average temptation | -.08 | [-.13;-.03] | -.27 | 3.21 | .002 | -.08 | [-.14;-.03] | -.28 | 3.08 | .003 | -.09 | [-.15;-.02] | -.26 | 2.63 | .010 |
| | F(2,133) = 5.95, p=.003, R2 = .083 | | | | | F(2,118) = 5.16, p=.007, R2 = .080 | | | | | F(2,97) = 4.19, p=.018, R2 = .079 | | | | |

Note. Table shows results from three standard multiple regression (two-tailed), one at each follow-up. P-values are uncorrected.

## 3. Analyses with conflicting desires only

We conducted further correlations to include three experience sampling variables for events where participants reported conflicting desires (i.e., desires that conflict at least somewhat with a goal, rather than all desires). These the analyses are reported in table S1 and include the following variables not included in the main manuscript:

- Desire strength for conflicting desires ("desirestrength" in the table below).
- Resistance to conflicting desires ("resistance" in the table below).
- proportion of desires given in to for conflicting desires only (i.e., "enactment" in the table below).

Crucially, these analyses did not meaningful relationships between ERPs related to self-regulation and variables related to conflicting desires.

Table S2: table of correlations including variables (desire strength, resistance, and enactment) relating to conflicting desires

**Bayesian Pearson Correlations**

| | | Pearson's r | $BF_{10}$ |
|---|---|---|---|
| extra | - resistance | -0.068 | 0.141 |
| extra | - desirestrength | 0.079 | 0.161 |
| extra | - enactment | 0.036 | 0.106 |
| agree | - resistance | -0.091 | 0.193 |
| agree | - desirestrength | 0.195 | 2.791 |
| agree | - enactment | -0.091 | 0.193 |
| consci | - resistance | -0.123 | 0.347 |
| consci | - desirestrength | 0.109 | 0.269 |
| consci | - enactment | -0.150 | 0.664 |
| neuro | - resistance | 0.043 | 0.111 |
| neuro | - desirestrength | -0.040 | 0.108 |
| neuro | - enactment | -0.040 | 0.109 |
| open | - resistance | -0.038 | 0.107 |
| open | - desirestrength | 0.008 | 0.094 |
| open | - enactment | -0.077 | 0.157 |
| Self-control | - resistance | -0.069 | 0.142 |
| Self-control | - desirestrength | 0.106 | 0.249 |
| Self-control | - enactment | -0.068 | 0.140 |
| BIS | - resistance | 0.039 | 0.108 |
| BIS | - desirestrength | 0.071 | 0.146 |
| BIS | - enactment | -0.104 | 0.238 |

**Bayesian Pearson Correlations**

| | | Pearson's r | $BF_{10}$ |
|---|---|---|---|
| BAS | - resistance | -0.029 | 0.102 |
| BAS | - desirestrength | 0.173 | 1.340 |
| BAS | - enactment | -0.041 | 0.109 |
| LPP HA Pleasant | - resistance | -0.016 | 0.100 |
| LPP HA Pleasant | - desirestrength | -0.001 | 0.097 |
| LPP HA Pleasant | - enactment | -0.023 | 0.102 |
| Delta_Arousal | - resistance | 0.007 | 0.098 |
| Delta_Arousal | - desirestrength | -0.068 | 0.141 |
| Delta_Arousal | - enactment | -0.159 | 0.753 |
| Delta_Valence | - resistance | -0.019 | 0.101 |
| Delta_Valence | - desirestrength | -0.056 | 0.125 |
| Delta_Valence | - enactment | 0.071 | 0.146 |
| FCz DERN | - resistance | 0.003 | 0.104 |
| FCz DERN | - desirestrength | 0.093 | 0.192 |
| FCz DERN | - enactment | -0.017 | 0.106 |
| FCz Diff RewP | - resistance | -0.074 | 0.152 |
| FCz Diff RewP | - desirestrength | 0.040 | 0.110 |
| FCz Diff RewP | - enactment | 0.196 | 2.199 |
| FCz RewP Correct | - resistance | -0.040 | 0.111 |
| FCz RewP Correct | - desirestrength | 0.094 | 0.199 |
| FCz Rewp Correct | - enactment | 0.133 | 0.407 |
| Flanker Diff RT | - resistance | -0.078 | 0.159 |
| Flanker Diff RT | - desirestrength | 0.112 | 0.264 |
| Flanker Diff RT | - enactment | 0.181 | 1.298 |
| Flanker Diff Accuracy | - resistance | 0.040 | 0.112 |
| Flanker Diff Accuracy | - desirestrength | 0.045 | 0.115 |
| Flanker Diff Accuracy | - enactment | 0.011 | 0.100 |
| 1 month prog. | - resistance | -0.061 | 0.139 |
| 1 month prog. | - desirestrength | 0.076 | 0.158 |
| 1 month prog. | - enactment | 0.138 | 0.370 |
| 3 month prog. | - resistance | -0.027 | 0.119 |
| 3 month prog. | - desirestrength | 0.029 | 0.119 |
| 3 month prog. | - enactment | 0.230 | 2.637 |
| 6 month prog. | - resistance | -0.014 | 0.127 |
| 6 month prog. | - desirestrength | 0.162 | 0.453 |
| 6 month prog. | - enactment | 0.057 | 0.147 |
| resistance | - desirestrength | 0.049 | 0.116 |
| resistance | - enactment | -0.169 | 1.135 |
| desirestrength | - enactment | -0.007 | 0.095 |

Note. ERN: error related negativity, CRN: correct related negativity; DERN: ERN difference wave; LPP: Late Positive Potential; RewP: Reward Positivity; HA: High Arousal; LA: Low Arousal; BIS: Behavioural Inhibition System; BAS: Behavioural Activation System; Extra,

agree, consci, neuro, and open: Big-5 extraversion, agreeableness, conscientiousness, neuroticism, and openness, respectively; Self-control: Trait self-control; BF: Bayes Factor.

4. **Alternate operationalizations of ERPs (i.e., ERN, RewP, LPP).**

The analyses presented in the manuscript are operationalisations of the ERP results that are one of many possible variables extracted from the ERPs. For example, in addition to the difference ERN (error-correct), it is equally possible to explore correlations between the CRN and ERN separately; the LPP can be operationalised as early and late components for multiple different image types (high arousal positive, high arousal negative, low arousal positive, low arousal negative), and the RewP could also be presented with its error trial equivalent. We present Bayesian correlation pairs for each of these separate ERPs with each other person-level variable that we included in our study (see table S2). While ERPs were sometimes correlated with each other, we found no evidence that the ERPs were correlated with other forms of self-regulation such as traits, everyday experience sampling, or goal progress). Thus, these additional analyses did not alter the conclusions of the analyses presented in the main manuscript.

**Table S3: Bayesian Pearson Correlations including additional ERPs**

| | | Pearson's r | $BF_{10}$ |
|---|---|---|---|
| Flanker Diff RT | - LPP HA Pleasant | 1.169e -4 | 0.096 |
| Flanker Diff RT | - LPP LA Pleasant | -0.007 | 0.097 |
| Flanker Diff RT | - LPP HA Unpleasant | 0.080 | 0.163 |
| Flanker Diff RT | - LPP LA Unpleasant | 0.007 | 0.096 |
| Flanker Diff RT | - LPP Neutral | -0.010 | 0.097 |
| Flanker Diff RT | - Late LPP HA Pleasant | -0.076 | 0.155 |
| Flanker Diff RT | - Late LPP LA Pleasant | -0.056 | 0.124 |
| Flanker Diff RT | - Late LPP HA Unpleasant | -0.010 | 0.097 |
| Flanker Diff RT | - Late LPP LA Unpleasant | -0.020 | 0.099 |
| Flanker Diff RT | - Late LPP Neutral | -0.042 | 0.111 |
| Flanker Diff RT | - FCz ERN | 0.020 | 0.100 |
| Flanker Diff RT | - FCz DERN | 0.056 | 0.125 |
| Flanker Diff RT | - FCz CRN | -0.070 | 0.144 |
| Flanker Diff RT | - RewP Correct | -0.046 | 0.114 |
| Flanker Diff RT | - RewP Error | -0.028 | 0.103 |
| Flanker Diff RT | - RewP Difference | -0.041 | 0.110 |

| | | Pearson's r | $BF_{10}$ |
|---|---|---|---|
| Flanker Diff RT | - Delta_Arousal | 0.020 | 0.100 |
| Flanker Diff RT | - Delta_Valence | -0.089 | 0.186 |
| Flanker Diff ERs | - LPP HA Pleasant | 0.023 | 0.100 |
| Flanker Diff ERs | - LPP LA Pleasant | -0.056 | 0.124 |
| Flanker Diff ERs | - LPP HA Unpleasant | 0.016 | 0.098 |
| Flanker Diff ERs | - LPP LA Unpleasant | 0.002 | 0.096 |
| Flanker Diff ERs | - LPP Neutral | -0.010 | 0.096 |
| Flanker Diff ERs | - Late LPP HA Pleasant | 0.026 | 0.101 |
| Flanker Diff ERs | - Late LPP LA Pleasant | -0.054 | 0.122 |
| Flanker Diff ERs | - Late LPP HA Unpleasant | 0.082 | 0.167 |
| Flanker Diff ERs | - Late LPP LA Unpleasant | 0.016 | 0.098 |
| Flanker Diff ERs | - Late LPP Neutral | -0.001 | 0.096 |
| Flanker Diff ERs | - FCz ERN | 0.459 | 1.993e +7 |
| Flanker Diff ERs | - FCz DERN | 0.353 | 4750.121 |
| Flanker Diff ERs | - FCz CRN | 0.095 | 0.203 |
| Flanker Diff ERs | - RewP Correct | -0.068 | 0.142 |
| Flanker Diff ERs | - RewP Error | -0.068 | 0.142 |
| Flanker Diff ERs | - RewP Difference | -0.021 | 0.100 |
| Flanker Diff ERs | - Delta_Arousal | 0.101 | 0.225 |
| Flanker Diff ERs | - Delta_Valence | -0.083 | 0.171 |
| desire strength (all) | - LPP HA Pleasant | 0.006 | 0.093 |
| desire strength (all) | - LPP LA Pleasant | 0.057 | 0.124 |
| desire strength (all) | - LPP HA Unpleasant | 0.087 | 0.181 |
| desire strength (all) | - LPP LA Unpleasant | 0.091 | 0.193 |
| desire strength (all) | - LPP Neutral | 0.057 | 0.124 |
| desire strength (all) | - Late LPP HA Pleasant | -0.007 | 0.093 |
| desire strength (all) | - Late LPP LA Pleasant | 0.050 | 0.117 |
| desire strength (all) | - Late LPP HA Unpleasant | 0.068 | 0.140 |
| desire strength (all) | - Late LPP LA Unpleasant | 0.077 | 0.156 |
| desire strength (all) | - Late LPP Neutral | -0.036 | 0.104 |
| desire strength (all) | - FCz ERN | 0.013 | 0.100 |
| desire strength (all) | - FCz DERN | 0.101 | 0.218 |
| desire strength (all) | - FCz CRN | -0.189 | 1.833 |
| desire strength (all) | - RewP Correct | 0.100 | 0.227 |
| desire strength (all) | - RewP Error | 0.082 | 0.169 |
| desire strength (all) | - RewP Difference | 0.059 | 0.127 |
| desire strength (all) | - Delta_Arousal | -0.041 | 0.108 |
| desire strength (all) | - Delta_Valence | -0.106 | 0.250 |
| resistance (all) | - LPP HA Pleasant | -0.048 | 0.114 |
| resistance (all) | - LPP LA Pleasant | -0.132 | 0.434 |
| resistance (all) | - LPP HA Unpleasant | -0.052 | 0.118 |

| | | Pearson's r | $BF_{10}$ |
|---|---|---|---|
| resistance (all) | - LPP LA Unpleasant | -0.104 | 0.243 |
| resistance (all) | - LPP Neutral | -0.095 | 0.209 |
| resistance (all) | - Late LPP HA Pleasant | -0.105 | 0.247 |
| resistance (all) | - Late LPP LA Pleasant | -0.086 | 0.180 |
| resistance (all) | - Late LPP HA Unpleasant | -0.060 | 0.128 |
| resistance (all) | - Late LPP LA Unpleasant | -0.087 | 0.183 |
| resistance (all) | - Late LPP Neutral | -0.068 | 0.140 |
| resistance (all) | - FCz ERN | -0.022 | 0.103 |
| resistance (all) | - FCz DERN | 0.031 | 0.107 |
| resistance (all) | - FCz CRN | -0.071 | 0.147 |
| resistance (all) | - RewP Correct | -0.040 | 0.107 |
| resistance (all) | - RewP Error | -0.003 | 0.093 |
| resistance (all) | - RewP Difference | -0.067 | 0.139 |
| resistance (all) | - Delta_Arousal | 0.048 | 0.114 |
| resistance (all) | - Delta_Valence | -0.045 | 0.111 |
| prop desires | - LPP HA Pleasant | -0.079 | 0.161 |
| prop desires | - LPP LA Pleasant | -0.070 | 0.144 |
| prop desires | - LPP HA Unpleasant | -0.021 | 0.096 |
| prop desires | - LPP LA Unpleasant | -0.031 | 0.102 |
| prop desires | - LPP Neutral | -0.043 | 0.110 |
| prop desires | - Late LPP HA Pleasant | -0.059 | 0.127 |
| prop desires | - Late LPP LA Pleasant | -0.038 | 0.106 |
| prop desires | - Late LPP HA Unpleasant | 0.074 | 0.151 |
| prop desires | - Late LPP LA Unpleasant | 0.014 | 0.095 |
| prop desires | - Late LPP Neutral | -0.048 | 0.114 |
| prop desires | - FCz ERN | 0.001 | 0.099 |
| prop desires | - FCz DERN | 0.055 | 0.125 |
| prop desires | - FCz CRN | -0.083 | 0.170 |
| prop desires | - RewP Correct | -0.096 | 0.210 |
| prop desires | - RewP Error | -0.072 | 0.147 |
| prop desires | - RewP Difference | -0.066 | 0.136 |
| prop desires | - Delta_Arousal | 0.035 | 0.104 |
| prop desires | - Delta_Valence | -0.141 | 0.548 |
| desires enactment | - LPP HA Pleasant | -0.109 | 0.268 |
| desires enactment | - LPP LA Pleasant | -0.011 | 0.095 |
| desires enactment | - LPP HA Unpleasant | -0.074 | 0.152 |
| desires enactment | - LPP LA Unpleasant | -0.013 | 0.095 |
| desires enactment | - LPP Neutral | -0.041 | 0.108 |
| desires enactment | - Late LPP HA Pleasant | -0.089 | 0.189 |
| desires enactment | - Late LPP LA Pleasant | -0.056 | 0.124 |
| desires enactment | - Late LPP HA Unpleasant | -0.112 | 0.286 |

| | | Pearson's r | $BF_{10}$ |
|---|---|---|---|
| desires enactment | - Late LPP LA Unpleasant | -0.057 | 0.125 |
| desires enactment | - Late LPP Neutral | -0.092 | 0.196 |
| desires enactment | - FCz ERN | -0.012 | 0.100 |
| desires enactment | - FCz DERN | -0.011 | 0.100 |
| desires enactment | - FCz CRN | -0.014 | 0.099 |
| desires enactment | - RewP Correct | 0.061 | 0.129 |
| desires enactment | - RewP Error | 1.868e -4 | 0.094 |
| desires enactment | - RewP Difference | 0.111 | 0.275 |
| desires enactment | - Delta_Arousal | -0.122 | 0.348 |
| desires enactment | - Delta_Valence | 0.005 | 0.094 |
| avg_newdesstrength | - LPP HA Pleasant | -0.052 | 0.118 |
| avg_newdesstrength | - LPP LA Pleasant | -0.026 | 0.099 |
| avg_newdesstrength | - LPP HA Unpleasant | 0.017 | 0.095 |
| avg_newdesstrength | - LPP LA Unpleasant | 0.015 | 0.095 |
| avg_newdesstrength | - LPP Neutral | -0.013 | 0.094 |
| avg_newdesstrength | - Late LPP HA Pleasant | -0.043 | 0.110 |
| avg_newdesstrength | - Late LPP LA Pleasant | -0.005 | 0.094 |
| avg_newdesstrength | - Late LPP HA Unpleasant | 0.089 | 0.189 |
| avg_newdesstrength | - Late LPP LA Unpleasant | 0.049 | 0.115 |
| avg_newdesstrength | - Late LPP Neutral | -0.050 | 0.115 |
| avg_newdesstrength | - FCz ERN | -0.003 | 0.099 |
| avg_newdesstrength | - FCz DERN | 0.065 | 0.137 |
| avg_newdesstrength | - FCz CRN | -0.121 | 0.325 |
| avg_newdesstrength | - RewP Correct | -0.045 | 0.111 |
| avg_newdesstrength | - RewP Error | -0.037 | 0.105 |
| avg_newdesstrength | - RewP Difference | -0.026 | 0.099 |
| avg_newdesstrength | - Delta_Arousal | 0.014 | 0.095 |
| avg_newdesstrength | - Delta_Valence | -0.149 | 0.682 |
| extra | - LPP HA Pleasant | 0.038 | 0.104 |
| extra | - LPP LA Pleasant | 0.036 | 0.103 |
| extra | - LPP HA Unpleasant | 0.110 | 0.280 |
| extra | - LPP LA Unpleasant | 0.084 | 0.175 |
| extra | - LPP Neutral | -0.004 | 0.091 |
| extra | - Late LPP HA Pleasant | 0.055 | 0.121 |
| extra | - Late LPP LA Pleasant | 0.046 | 0.111 |
| extra | - Late LPP HA Unpleasant | 0.124 | 0.380 |
| extra | - Late LPP LA Unpleasant | 0.029 | 0.098 |
| extra | - Late LPP Neutral | 0.007 | 0.092 |
| extra | - FCz ERN | 0.016 | 0.099 |
| extra | - FCz DERN | 0.085 | 0.174 |
| extra | - FCz CRN | -0.112 | 0.275 |

| | | Pearson's r | $BF_{10}$ |
|---|---|---|---|
| extra | - RewP Correct | 0.029 | 0.099 |
| extra | - RewP Error | 0.016 | 0.094 |
| extra | - RewP Difference | 0.028 | 0.099 |
| extra | - Delta_Arousal | 0.063 | 0.132 |
| extra | - Delta_Valence | -0.078 | 0.161 |
| agree | - LPP HA Pleasant | 0.090 | 0.192 |
| agree | - LPP LA Pleasant | 0.075 | 0.153 |
| agree | - LPP HA Unpleasant | 0.117 | 0.324 |
| agree | - LPP LA Unpleasant | 0.158 | 0.947 |
| agree | - LPP Neutral | 0.171 | 1.415 |
| agree | - Late LPP HA Pleasant | 0.112 | 0.292 |
| agree | - Late LPP LA Pleasant | 0.037 | 0.104 |
| agree | - Late LPP HA Unpleasant | 0.116 | 0.319 |
| agree | - Late LPP LA Unpleasant | 0.128 | 0.422 |
| agree | - Late LPP Neutral | 0.122 | 0.364 |
| agree | - FCz ERN | 0.119 | 0.310 |
| agree | - FCz DERN | 0.135 | 0.431 |
| agree | - FCz CRN | -0.074 | 0.151 |
| agree | - RewP Correct | 0.043 | 0.108 |
| agree | - RewP Error | 0.023 | 0.096 |
| agree | - RewP Difference | 0.043 | 0.108 |
| agree | - Delta_Arousal | 0.021 | 0.095 |
| agree | - Delta_Valence | -0.084 | 0.176 |
| consci | - LPP HA Pleasant | -0.071 | 0.146 |
| consci | - LPP LA Pleasant | -0.005 | 0.092 |
| consci | - LPP HA Unpleasant | 0.015 | 0.093 |
| consci | - LPP LA Unpleasant | -0.009 | 0.092 |
| consci | - LPP Neutral | 0.019 | 0.094 |
| consci | - Late LPP HA Pleasant | -0.071 | 0.145 |
| consci | - Late LPP LA Pleasant | -0.055 | 0.121 |
| consci | - Late LPP HA Unpleasant | -0.009 | 0.092 |
| consci | - Late LPP LA Unpleasant | -0.025 | 0.096 |
| consci | - Late LPP Neutral | -0.046 | 0.111 |
| consci | - FCz ERN | 0.042 | 0.112 |
| consci | - FCz DERN | -0.061 | 0.131 |
| consci | - FCz CRN | 0.174 | 1.296 |
| consci | - RewP Correct | -0.035 | 0.103 |
| consci | - RewP Error | 0.023 | 0.096 |
| consci | - RewP Difference | -0.099 | 0.225 |
| consci | - Delta_Arousal | -0.043 | 0.108 |
| consci | - Delta_Valence | -0.062 | 0.131 |

| | | Pearson's r | $BF_{10}$ |
|---|---|---|---|
| neuro | - LPP HA Pleasant | 0.020 | 0.094 |
| neuro | - LPP LA Pleasant | -0.023 | 0.096 |
| neuro | - LPP HA Unpleasant | -0.040 | 0.106 |
| neuro | - LPP LA Unpleasant | -0.018 | 0.094 |
| neuro | - LPP Neutral | 0.018 | 0.094 |
| neuro | - Late LPP HA Pleasant | 0.051 | 0.116 |
| neuro | - Late LPP LA Pleasant | 0.060 | 0.128 |
| neuro | - Late LPP HA Unpleasant | -0.027 | 0.097 |
| neuro | - Late LPP LA Unpleasant | 0.012 | 0.093 |
| neuro | - Late LPP Neutral | 0.002 | 0.091 |
| neuro | - FCz ERN | 0.040 | 0.110 |
| neuro | - FCz DERN | 0.092 | 0.193 |
| neuro | - FCz CRN | -0.074 | 0.151 |
| neuro | - RewP Correct | -0.022 | 0.096 |
| neuro | - RewP Error | -0.039 | 0.106 |
| neuro | - RewP Difference | 0.019 | 0.095 |
| neuro | - Delta_Arousal | 0.008 | 0.092 |
| neuro | - Delta_Valence | 0.068 | 0.140 |
| open | - LPP HA Pleasant | 0.045 | 0.110 |
| open | - LPP LA Pleasant | -0.006 | 0.092 |
| open | - LPP HA Unpleasant | 0.019 | 0.094 |
| open | - LPP LA Unpleasant | 0.033 | 0.101 |
| open | - LPP Neutral | 0.028 | 0.098 |
| open | - Late LPP HA Pleasant | 0.062 | 0.130 |
| open | - Late LPP LA Pleasant | -0.009 | 0.092 |
| open | - Late LPP HA Unpleasant | 0.006 | 0.091 |
| open | - Late LPP LA Unpleasant | -0.018 | 0.094 |
| open | - Late LPP Neutral | -0.018 | 0.094 |
| open | - FCz ERN | 0.013 | 0.098 |
| open | - FCz DERN | 0.009 | 0.097 |
| open | - FCz CRN | 0.019 | 0.098 |
| open | - RewP Correct | 0.083 | 0.173 |
| open | - RewP Error | 0.072 | 0.147 |
| open | - RewP Difference | 0.042 | 0.108 |
| open | - Delta_Arousal | 0.061 | 0.129 |
| open | - Delta_Valence | 0.027 | 0.098 |
| BIS | - LPP HA Pleasant | 0.045 | 0.110 |
| BIS | - LPP LA Pleasant | -0.059 | 0.126 |
| BIS | - LPP HA Unpleasant | 0.019 | 0.094 |
| BIS | - LPP LA Unpleasant | -0.019 | 0.094 |
| BIS | - LPP Neutral | 0.025 | 0.097 |

| | | Pearson's r | $BF_{10}$ |
|---|---|---|---|
| BIS | - Late LPP HA Pleasant | 0.067 | 0.137 |
| BIS | - Late LPP LA Pleasant | -0.020 | 0.095 |
| BIS | - Late LPP HA Unpleasant | 0.044 | 0.109 |
| BIS | - Late LPP LA Unpleasant | -0.028 | 0.098 |
| BIS | - Late LPP Neutral | -0.015 | 0.093 |
| BIS | - FCz ERN | 0.045 | 0.114 |
| BIS | - FCz DERN | 0.141 | 0.500 |
| BIS | - FCz CRN | -0.179 | 1.473 |
| BIS | - RewP Correct | 0.062 | 0.130 |
| BIS | - RewP Error | 0.026 | 0.097 |
| BIS | - RewP Difference | 0.072 | 0.147 |
| BIS | - Delta_Arousal | 0.130 | 0.440 |
| BIS | - Delta_Valence | 0.002 | 0.092 |
| BAS | - LPP HA Pleasant | 0.032 | 0.100 |
| BAS | - LPP LA Pleasant | 0.037 | 0.103 |
| BAS | - LPP HA Unpleasant | 0.093 | 0.204 |
| BAS | - LPP LA Unpleasant | 0.046 | 0.111 |
| BAS | - LPP Neutral | -0.012 | 0.092 |
| BAS | - Late LPP HA Pleasant | 0.065 | 0.135 |
| BAS | - Late LPP LA Pleasant | 0.013 | 0.093 |
| BAS | - Late LPP HA Unpleasant | 0.086 | 0.182 |
| BAS | - Late LPP LA Unpleasant | 0.037 | 0.103 |
| BAS | - Late LPP Neutral | -0.020 | 0.094 |
| BAS | - FCz ERN | -0.029 | 0.104 |
| BAS | - FCz DERN | 0.066 | 0.138 |
| BAS | - FCz CRN | -0.153 | 0.707 |
| BAS | - RewP Correct | 0.121 | 0.352 |
| BAS | - RewP Error | 0.090 | 0.194 |
| BAS | - RewP Difference | 0.082 | 0.171 |
| BAS | - Delta_Arousal | 0.057 | 0.124 |
| BAS | - Delta_Valence | -0.039 | 0.105 |
| self-control | - LPP HA Pleasant | 0.022 | 0.095 |
| self-control | - LPP LA Pleasant | 0.084 | 0.174 |
| self-control | - LPP HA Unpleasant | 0.066 | 0.136 |
| self-control | - LPP LA Unpleasant | 0.047 | 0.112 |
| self-control | - LPP Neutral | 0.022 | 0.095 |
| self-control | - Late LPP HA Pleasant | 0.003 | 0.091 |
| self-control | - Late LPP LA Pleasant | 0.031 | 0.100 |
| self-control | - Late LPP HA Unpleasant | 0.040 | 0.106 |
| self-control | - Late LPP LA Unpleasant | 0.012 | 0.092 |
| self-control | - Late LPP Neutral | -0.032 | 0.100 |

| | | Pearson's r | $BF_{10}$ |
|---|---|---|---|
| self-control | - FCz ERN | 0.077 | 0.158 |
| self-control | - FCz DERN | -0.019 | 0.100 |
| self-control | - FCz CRN | 0.152 | 0.689 |
| self-control | - RewP Correct | -0.041 | 0.107 |
| self-control | - RewP Error | -0.033 | 0.101 |
| self-control | - RewP Difference | -0.025 | 0.097 |
| self-control | - Delta_Arousal | -0.021 | 0.095 |
| self-control | - Delta_Valence | 0.003 | 0.092 |
| self-control | - 1 month prog. | 0.226 | 4.568 |
| self-control | - 3 month prog. | 0.218 | 2.434 |
| self-control | - 6 month prog. | 0.278 | 7.586 |
| LPP HA Pleasant | - LPP LA Pleasant | 0.704 | 3.816e +26 |
| LPP HA Pleasant | - LPP HA Unpleasant | 0.803 | 2.022e +41 |
| LPP HA Pleasant | - LPP LA Unpleasant | 0.770 | 2.600e +35 |
| LPP HA Pleasant | - LPP Neutral | 0.737 | 9.909e +30 |
| LPP HA Pleasant | - Late LPP HA Pleasant | 0.789 | 4.891e +38 |
| LPP HA Pleasant | - Late LPP LA Pleasant | 0.588 | 1.207e +16 |
| LPP HA Pleasant | - Late LPP HA Unpleasant | 0.644 | 6.277e +20 |
| LPP HA Pleasant | - Late LPP LA Unpleasant | 0.600 | 1.119e +17 |
| LPP HA Pleasant | - Late LPP Neutral | 0.609 | 7.697e +17 |
| LPP HA Pleasant | - FCz ERN | 0.146 | 0.571 |
| LPP HA Pleasant | - FCz DERN | 0.094 | 0.201 |
| LPP HA Pleasant | - FCz CRN | 0.048 | 0.115 |
| LPP HA Pleasant | - RewP Correct | 0.130 | 0.443 |
| LPP HA Pleasant | - RewP Error | 0.036 | 0.103 |
| LPP HA Pleasant | - RewP Difference | 0.182 | 2.010 |
| LPP HA Pleasant | - Delta_Arousal | 0.424 | 9.272e +6 |
| LPP HA Pleasant | - Delta_Valence | 0.092 | 0.200 |
| LPP HA Pleasant | - 1 month prog. | 0.133 | 0.362 |
| LPP HA Pleasant | - 3 month prog. | 0.047 | 0.128 |
| LPP HA Pleasant | - 6 month prog. | 0.070 | 0.158 |
| LPP LA Pleasant | - LPP HA Unpleasant | 0.724 | 9.149e +28 |
| LPP LA Pleasant | - LPP LA Unpleasant | 0.787 | 9.873e +37 |
| LPP LA Pleasant | - LPP Neutral | 0.786 | 6.102e +37 |
| LPP LA Pleasant | - Late LPP HA Pleasant | 0.507 | 9.091e +10 |
| LPP LA Pleasant | - Late LPP LA Pleasant | 0.841 | 3.894e +48 |
| LPP LA Pleasant | - Late LPP HA Unpleasant | 0.544 | 1.300e +13 |
| LPP LA Pleasant | - Late LPP LA Unpleasant | 0.643 | 3.130e +20 |
| LPP LA Pleasant | - Late LPP Neutral | 0.651 | 1.570e +21 |
| LPP LA Pleasant | - FCz ERN | 0.051 | 0.120 |
| LPP LA Pleasant | - FCz DERN | -0.019 | 0.100 |

| | | Pearson's r | $BF_{10}$ |
|---|---|---|---|
| LPP LA Pleasant | - FCz CRN | 0.110 | 0.266 |
| LPP LA Pleasant | - RewP Correct | 0.085 | 0.176 |
| LPP LA Pleasant | - RewP Error | 0.057 | 0.124 |
| LPP LA Pleasant | - RewP Difference | 0.066 | 0.137 |
| LPP LA Pleasant | - Delta_Arousal | -0.182 | 2.066 |
| LPP LA Pleasant | - Delta_Valence | 0.119 | 0.345 |
| LPP LA Pleasant | - 1 month prog. | 0.233 | 4.972 |
| LPP LA Pleasant | - 3 month prog. | 0.153 | 0.471 |
| LPP LA Pleasant | - 6 month prog. | 0.241 | 2.339 |
| LPP HA Unpleasant | - LPP LA Unpleasant | 0.791 | 6.508e +38 |
| LPP HA Unpleasant | - LPP Neutral | 0.746 | 1.625e +32 |
| LPP HA Unpleasant | - Late LPP HA Pleasant | 0.527 | 1.805e +12 |
| LPP HA Unpleasant | - Late LPP LA Pleasant | 0.534 | 3.372e +12 |
| LPP HA Unpleasant | - Late LPP HA Unpleasant | 0.800 | 5.669e +40 |
| LPP HA Unpleasant | - Late LPP LA Unpleasant | 0.617 | 2.397e +18 |
| LPP HA Unpleasant | - Late LPP Neutral | 0.589 | 2.268e +16 |
| LPP HA Unpleasant | - FCz ERN | 0.085 | 0.175 |
| LPP HA Unpleasant | - FCz DERN | 0.054 | 0.123 |
| LPP HA Unpleasant | - FCz CRN | 0.038 | 0.107 |
| LPP HA Unpleasant | - RewP Correct | 0.134 | 0.484 |
| LPP HA Unpleasant | - RewP Error | 0.065 | 0.135 |
| LPP HA Unpleasant | - RewP Difference | 0.144 | 0.635 |
| LPP HA Unpleasant | - Delta_Arousal | 0.364 | 50167.557 |
| LPP HA Unpleasant | - Delta_Valence | -0.324 | 2746.193 |
| LPP HA Unpleasant | - 1 month prog. | 0.155 | 0.563 |
| LPP HA Unpleasant | - 3 month prog. | 0.003 | 0.112 |
| LPP HA Unpleasant | - 6 month prog. | 0.056 | 0.144 |
| LPP LA Unpleasant | - LPP Neutral | 0.780 | 1.095e +37 |
| LPP LA Unpleasant | - Late LPP HA Pleasant | 0.515 | 2.845e +11 |
| LPP LA Unpleasant | - Late LPP LA Pleasant | 0.602 | 1.319e +17 |
| LPP LA Unpleasant | - Late LPP HA Unpleasant | 0.617 | 2.826e +18 |
| LPP LA Unpleasant | - Late LPP LA Unpleasant | 0.837 | 8.453e +47 |
| LPP LA Unpleasant | - Late LPP Neutral | 0.633 | 5.249e +19 |
| LPP LA Unpleasant | - FCz ERN | 0.132 | 0.404 |
| LPP LA Unpleasant | - FCz DERN | 0.083 | 0.170 |
| LPP LA Unpleasant | - FCz CRN | 0.049 | 0.117 |
| LPP LA Unpleasant | - RewP Correct | 0.033 | 0.101 |
| LPP LA Unpleasant | - RewP Error | -0.020 | 0.095 |
| LPP LA Unpleasant | - RewP Difference | 0.089 | 0.190 |
| LPP LA Unpleasant | - Delta_Arousal | -0.053 | 0.118 |
| LPP LA Unpleasant | - Delta_Valence | -0.301 | 632.250 |

| | | Pearson's r | $BF_{10}$ |
|---|---|---|---|
| LPP LA Unpleasant | - 1 month prog. | 0.145 | 0.459 |
| LPP LA Unpleasant | - 3 month prog. | 0.039 | 0.123 |
| LPP LA Unpleasant | - 6 month prog. | 0.158 | 0.426 |
| LPP Neutral | - Late LPP HA Pleasant | 0.549 | 4.027e +13 |
| LPP Neutral | - Late LPP LA Pleasant | 0.641 | 2.053e +20 |
| LPP Neutral | - Late LPP HA Unpleasant | 0.591 | 2.958e +16 |
| LPP Neutral | - Late LPP LA Unpleasant | 0.646 | 7.532e +20 |
| LPP Neutral | - Late LPP Neutral | 0.842 | 1.829e +49 |
| LPP Neutral | - FCz ERN | 0.082 | 0.169 |
| LPP Neutral | - FCz DERN | -0.002 | 0.096 |
| LPP Neutral | - FCz CRN | 0.127 | 0.381 |
| LPP Neutral | - RewP Correct | 0.126 | 0.399 |
| LPP Neutral | - RewP Error | 0.082 | 0.170 |
| LPP Neutral | - RewP Difference | 0.104 | 0.249 |
| LPP Neutral | - Delta_Arousal | 0.046 | 0.111 |
| LPP Neutral | - Delta_Valence | -0.050 | 0.114 |
| LPP Neutral | - 1 month prog. | 0.204 | 2.024 |
| LPP Neutral | - 3 month prog. | 0.028 | 0.117 |
| LPP Neutral | - 6 month prog. | 0.152 | 0.391 |
| Late LPP HA Pleasant | - Late LPP LA Pleasant | 0.608 | 4.057e +17 |
| Late LPP HA Pleasant | - Late LPP HA Unpleasant | 0.601 | 1.775e +17 |
| Late LPP HA Pleasant | - Late LPP LA Unpleasant | 0.544 | 1.534e +13 |
| Late LPP HA Pleasant | - Late LPP Neutral | 0.600 | 1.521e +17 |
| Late LPP HA Pleasant | - FCz ERN | 0.111 | 0.270 |
| Late LPP HA Pleasant | - FCz DERN | 0.064 | 0.134 |
| Late LPP HA Pleasant | - FCz CRN | 0.054 | 0.121 |
| Late LPP HA Pleasant | - RewP Correct | 0.101 | 0.233 |
| Late LPP HA Pleasant | - RewP Error | 0.005 | 0.092 |
| Late LPP HA Pleasant | - RewP Difference | 0.173 | 1.509 |
| Late LPP HA Pleasant | - Delta_Arousal | 0.467 | 8.072e +8 |
| Late LPP HA Pleasant | - Delta_Valence | 0.319 | 1975.287 |
| Late LPP HA Pleasant | - 1 month prog. | 0.014 | 0.106 |
| Late LPP HA Pleasant | - 3 month prog. | -0.042 | 0.124 |
| Late LPP HA Pleasant | - 6 month prog. | 0.042 | 0.135 |
| Late LPP LA Pleasant | - Late LPP HA Unpleasant | 0.567 | 4.232e +14 |
| Late LPP LA Pleasant | - Late LPP LA Unpleasant | 0.652 | 2.172e +21 |
| Late LPP LA Pleasant | - Late LPP Neutral | 0.689 | 9.722e +24 |
| Late LPP LA Pleasant | - FCz ERN | 0.042 | 0.112 |
| Late LPP LA Pleasant | - FCz DERN | -0.007 | 0.097 |
| Late LPP LA Pleasant | - FCz CRN | 0.075 | 0.154 |
| Late LPP LA Pleasant | - RewP Correct | 0.049 | 0.114 |

| | | Pearson's r | $BF_{10}$ |
|---|---|---|---|
| Late LPP LA Pleasant | - RewP Error | 0.003 | 0.092 |
| Late LPP LA Pleasant | - RewP Difference | 0.082 | 0.171 |
| Late LPP LA Pleasant | - Delta_Arousal | -0.196 | 3.449 |
| Late LPP LA Pleasant | - Delta_Valence | 0.277 | 151.909 |
| Late LPP LA Pleasant | - 1 month prog. | 0.125 | 0.314 |
| Late LPP LA Pleasant | - 3 month prog. | 0.161 | 0.544 |
| Late LPP LA Pleasant | - 6 month prog. | 0.236 | 2.036 |
| Late LPP HA Unpleasant | - Late LPP LA Unpleasant | 0.679 | 1.161e +24 |
| Late LPP HA Unpleasant | - Late LPP Neutral | 0.611 | 1.145e +18 |
| Late LPP HA Unpleasant | - FCz ERN | 0.133 | 0.424 |
| Late LPP HA Unpleasant | - FCz DERN | 0.099 | 0.217 |
| Late LPP HA Unpleasant | - FCz CRN | 0.030 | 0.102 |
| Late LPP HA Unpleasant | - RewP Correct | 0.059 | 0.126 |
| Late LPP HA Unpleasant | - RewP Error | -0.032 | 0.100 |
| Late LPP HA Unpleasant | - RewP Difference | 0.155 | 0.850 |
| Late LPP HA Unpleasant | - Delta_Arousal | 0.427 | 1.173e +7 |
| Late LPP HA Unpleasant | - Delta_Valence | -0.372 | 91095.653 |
| Late LPP HA Unpleasant | - 1 month prog. | 0.053 | 0.127 |
| Late LPP HA Unpleasant | - 3 month prog. | 0.026 | 0.117 |
| Late LPP HA Unpleasant | - 6 month prog. | 0.026 | 0.128 |
| Late LPP LA Unpleasant | - Late LPP Neutral | 0.701 | 2.406e +26 |
| Late LPP LA Unpleasant | - FCz ERN | 0.129 | 0.380 |
| Late LPP LA Unpleasant | - FCz DERN | 0.102 | 0.230 |
| Late LPP LA Unpleasant | - FCz CRN | 0.015 | 0.097 |
| Late LPP LA Unpleasant | - RewP Correct | -0.015 | 0.093 |
| Late LPP LA Unpleasant | - RewP Error | -0.086 | 0.182 |
| Late LPP LA Unpleasant | - RewP Difference | 0.103 | 0.245 |
| Late LPP LA Unpleasant | - Delta_Arousal | -0.131 | 0.453 |
| Late LPP LA Unpleasant | - Delta_Valence | -0.350 | 17897.549 |
| Late LPP LA Unpleasant | - 1 month prog. | 0.027 | 0.110 |
| Late LPP LA Unpleasant | - 3 month prog. | 0.015 | 0.114 |
| Late LPP LA Unpleasant | - 6 month prog. | 0.155 | 0.410 |
| Late LPP Neutral | - FCz ERN | 0.093 | 0.197 |
| Late LPP Neutral | - FCz DERN | 0.032 | 0.105 |
| Late LPP Neutral | - FCz CRN | 0.090 | 0.189 |
| Late LPP Neutral | - RewP Correct | 0.083 | 0.172 |
| Late LPP Neutral | - RewP Error | 0.004 | 0.091 |
| Late LPP Neutral | - RewP Difference | 0.143 | 0.619 |
| Late LPP Neutral | - Delta_Arousal | 0.016 | 0.093 |
| Late LPP Neutral | - Delta_Valence | -0.008 | 0.091 |
| Late LPP Neutral | - 1 month prog. | 0.097 | 0.203 |

| | | Pearson's r | $BF_{10}$ |
|---|---|---|---|
| Late LPP Neutral | - 3 month prog. | 0.006 | 0.112 |
| Late LPP Neutral | - 6 month prog. | 0.119 | 0.249 |
| FCz ERN | - FCz DERN | 0.839 | 5.795e +42 |
| FCz ERN | - FCz CRN | 0.043 | 0.112 |
| FCz ERN | - RewP Correct | -0.123 | 0.334 |
| FCz ERN | - RewP Error | -0.148 | 0.596 |
| FCz ERN | - RewP Difference | 0.001 | 0.097 |
| FCz ERN | - Delta_Arousal | 0.076 | 0.156 |
| FCz ERN | - Delta_Valence | -0.046 | 0.115 |
| FCz ERN | - 1 month prog. | 0.053 | 0.131 |
| FCz ERN | - 3 month prog. | 0.086 | 0.176 |
| FCz ERN | - 6 month prog. | 0.055 | 0.148 |
| FCz DERN | - FCz CRN | -0.509 | 5.940e +9 |
| FCz DERN | - RewP Correct | -0.075 | 0.153 |
| FCz DERN | - RewP Error | -0.102 | 0.227 |
| FCz DERN | - RewP Difference | 0.018 | 0.099 |
| FCz DERN | - Delta_Arousal | 0.081 | 0.165 |
| FCz DERN | - Delta_Valence | -0.084 | 0.173 |
| FCz DERN | - 1 month prog. | 0.064 | 0.142 |
| FCz DERN | - 3 month prog. | 0.068 | 0.152 |
| FCz DERN | - 6 month prog. | 0.082 | 0.175 |
| FCz CRN | - RewP Correct | -0.072 | 0.148 |
| FCz CRN | - RewP Error | -0.064 | 0.135 |
| FCz CRN | - RewP Difference | -0.034 | 0.105 |
| FCz CRN | - Delta_Arousal | -0.025 | 0.101 |
| FCz CRN | - Delta_Valence | 0.072 | 0.147 |
| FCz CRN | - 1 month prog. | -0.034 | 0.116 |
| FCz CRN | - 3 month prog. | 0.014 | 0.117 |
| FCz CRN | - 6 month prog. | -0.040 | 0.137 |
| RewP Correct | - RewP Error | 0.833 | 2.863e +46 |
| RewP Correct | - RewP Difference | 0.555 | 5.372e +13 |
| RewP Correct | - Delta_Arousal | 0.135 | 0.484 |
| RewP Correct | - Delta_Valence | 0.056 | 0.122 |
| RewP Correct | - 1 month prog. | 0.060 | 0.135 |
| RewP Correct | - 3 month prog. | 8.215e -4 | 0.112 |
| RewP Correct | - 6 month prog. | 0.041 | 0.135 |
| RewP Error | - RewP Difference | 0.001 | 0.091 |
| RewP Error | - Delta_Arousal | 0.054 | 0.120 |
| RewP Error | - Delta_Valence | 0.066 | 0.137 |
| RewP Error | - 1 month prog. | 0.039 | 0.117 |
| RewP Error | - 3 month prog. | 0.020 | 0.115 |

| | | Pearson's r | BF₁₀ |
|---|---|---|---|
| RewP Error | - 6 month prog. | 0.063 | 0.151 |
| RewP Difference | - Delta_Arousal | 0.160 | 0.982 |
| RewP Difference | - Delta_Valence | 5.791e -4 | 0.092 |
| RewP Difference | - 1 month prog. | 0.049 | 0.124 |
| RewP Difference | - 3 month prog. | -0.027 | 0.117 |
| RewP Difference | - 6 month prog. | -0.017 | 0.126 |
| Delta_Arousal | - Delta_Valence | -0.034 | 0.101 |
| Delta_Arousal | - 1 month prog. | -0.090 | 0.185 |
| Delta_Arousal | - 3 month prog. | -0.137 | 0.352 |
| Delta_Arousal | - 6 month prog. | -0.235 | 2.016 |
| Delta_Valence | - 1 month prog. | 0.072 | 0.150 |
| Delta_Valence | - 3 month prog. | 0.096 | 0.196 |
| Delta_Valence | - 6 month prog. | 0.103 | 0.209 |
| 1 month prog. | - 3 month prog. | 0.513 | 1.207e +7 |
| 1 month prog. | - 6 month prog. | 0.371 | 174.388 |
| 3 month prog. | - 6 month prog. | 0.527 | 1.089e +6 |

Note. ERN: error related negativity, CRN: correct related negativity; DERN: ERN difference wave; LPP: Late Positive Potential; RewP: Reward Positivity; HA: High Arousal; LA: Low Arousal; BIS: Behavioural Inhibition System; BAS: Behavioural Activation System; Extra, agree, consci, neuro, and open: Big-5 extraversion, agreeableness, conscientiousness, neuroticism, and openness, respectively; Self-control: Trait self-control; BF: Bayes Factor.
