# Supplementary material for: Longitudinal evidence that Event Related Potential measures of self-regulation do not predict everyday goal pursuit
Source: Nat Commun. 2022 Jun 9;13:3201. doi: 10.1038/s41467-022-30786-7 (PMC9184581; doi:10.1038/s41467-022-30786-7)
Supplement: Supplementary file 3 — Reporting Summary [file 41467_2022_30786_MOESM3_ESM.pdf]

nature portfolio

Corresponding author(s): Blair Saunders
Last updated by author(s): 19/04/22

Reporting Summary

Nature Portfolio wishes to improve the reproducibility of the work that we publish. This form provides structure for consistency and transparency in reporting. For further information on Nature Portfolio policies, see our Editorial Policies and the Editorial Policy Checklist.

Statistics

For all statistical analyses, confirm that the following items are present in the figure legend, table legend, main text, or Methods section.

n/a	Confirmed	
☐	☒	The exact sample size (*n*) for each experimental group/condition, given as a discrete number and unit of measurement
☐	☒	A statement on whether measurements were taken from distinct samples or whether the same sample was measured repeatedly
☐	☒	The statistical test(s) used AND whether they are one- or two-sided<br>*Only common tests should be described solely by name; describe more complex techniques in the Methods section.*
☒	☐	A description of all covariates tested
☐	☒	A description of any assumptions or corrections, such as tests of normality and adjustment for multiple comparisons
☐	☒	A full description of the statistical parameters including central tendency (e.g. means) or other basic estimates (e.g. regression coefficient) AND variation (e.g. standard deviation) or associated estimates of uncertainty (e.g. confidence intervals)
☐	☒	For null hypothesis testing, the test statistic (e.g. F, t, r) with confidence intervals, effect sizes, degrees of freedom and P value noted<br>*Give P values as exact values whenever suitable.*
☐	☒	For Bayesian analysis, information on the choice of priors and Markov chain Monte Carlo settings
☒	☐	For hierarchical and complex designs, identification of the appropriate level for tests and full reporting of outcomes
☐	☒	Estimates of effect sizes (e.g. Cohen's *d*, Pearson's *r*), indicating how they were calculated

Our web collection on statistics for biologists contains articles on many of the points above.

Software and code

Policy information about availability of computer code

Data collection	No bespoke computer code was used during data collection. Behavioural data was collected using MediaLab and Eprime 2.0 software. EEG data was collected using ASA acquisition software. SurveySignal was used to acquire the experience sampling data, Qualtrics was used to acquire all other survey software.
Data analysis	No bespoke computer code was used during data analysis. EEG data was analyzed using Brain Vision Analyzer v 2.2. All statistical analyses were conducted using JASP 0.14.1. Choice of priors for Bayesian analyses are presented in the method section.

For manuscripts utilizing custom algorithms or software that are central to the research but not yet described in published literature, software must be made available to editors and reviewers. We strongly encourage code deposition in a community repository (e.g. GitHub). See the Nature Portfolio guidelines for submitting code & software for further information.

Data

Policy information about availability of data

All manuscripts must include a data availability statement. This statement should provide the following information, where applicable:

- Accession codes, unique identifiers, or web links for publicly available datasets
- A description of any restrictions on data availability
- For clinical datasets or third party data, please ensure that the statement adheres to our policy

The data that support the findings of this study are available on request from the corresponding author. The data are not publicly available due to privacy or ethical restrictions.

March 2021

Field-specific reporting

Please select the one below that is the best fit for your research. If you are not sure, read the appropriate sections before making your selection.

☐ Life sciences ☒ Behavioural & social sciences ☐ Ecological, evolutionary & environmental sciences

For a reference copy of the document with all sections, see nature.com/documents/nr-reporting-summary-flat.pdf

Behavioural & social sciences study design

All studies must disclose on these points even when the disclosure is negative.

Study description	quantitative, cross-sectional design with mixed-methods (EEG, behaviour, experience sampling, self-reported personality and goal progress).
Research sample	Non-representative convenience sample of Individuals recruited from the University of Toronto Scarborough campus (92.4% students, 63% female, mean age 20.4 years, SD= 5.9). This sample was chosen due to the location of the EEG laboratory at the University of Toronto Scarborough.
Sampling strategy	A convenience sample of Participants who were predominantly recruited through an undergraduate participant pool, though a smaller number were also recruited through on-campus and local advertisements at the University of Toronto Scarborough. Although we did not conduct an a-priori power analysis, a sensitivity analysis showed that this sample size would allow us to find effects as small as r = .19 with 80% power for the between-subject analyses.
Data collection	Please see methods section for details of apparatus and materials. The participants completed the time one assessments in a separated chamber withing the EEG laboratory at the University of Toronto Scarborough that was adjacent to a room in which a research assistant monitored the incoming EEG and was available for questions. We used no blinding procedure. The EEG was recorded from 36 Ag/AgCl sintered electrodes arranged according to the international 10-20 system in a stretch-lycra cap (Electro-Cap International, Eton, OH). Vertical electro-oculography (VEOG) was recorded via a supra- to sub-orbital bipolar montage surrounding the right eye. The continuous EEG signal was digitized at 512 Hz using ASA acquisition hardware (asalab 4.9.4, TMSi Refa8 device; Advanced Neuro Technology, Enschede, the Netherlands). Reaction times were recorded MediaLab (v2012; Emiprisoft, New York, NY) using a a millisecond accurate QWERTY keyboard (Empirisoft DirectIN Millisecond Accurate Keyboard). Participants completed all follow-up surveys on their own personal device. We used no blinding procedure.
Timing	Baseline data collected from August 2014 to March 2017; ESM data collected 1 week following each participant's baseline (September 2014 to March 2017), follow-up date collected from each participant 1-, 3-, and 6- months following that participant's baseline.
Data exclusions	All data exclusions are reported throughout the manuscript and are variable depending on the analyses/timepoint. None of the exclusions were preestablished, but most were due to missing data.<br><br>At baseline, a total of 25 participants were excluded because they did not respond to at least 5 ESM signals and had no follow-up or usable EEG data.<br><br>214 participants had EEG recordings. 16 participants were excluded from the EEG analyses either because they had flat EEG signals (n=1) or because they had already been excluded from the study for reasons not pertaining to the EEG portion (n=15). Participants were then removed from EEG analyses on an ERP-by-ERP basis. ERN: Too-few usable trials (n = 7); more than 40% error on flanker (n = 11); no EEG data on Flanker task (n=10). RewP: having fewer than 20 usable RewP trials (n =3); having no triggers during feedback task (n=6). LPP: no image triggers (n = 4); too few cells > 8 trials to compute any LPP (n = 1); excluded from select cells because they had <8 usable LPP trials (n=3).<br><br>For the flanker task scores, 7 participants who did not complete it correctly and 13 participants with >40% error were excluded.<br><br>For the follow-ups, after removing duplicate and blank entries (i.e., participants who clicked on the survey but did not complete any of it), participants were excluded if they had incomplete goal progress data (n=6 at follow-up 1, n=4 at follow-up 2, n=3 at follow-up 3). Number of participants reported in the manuscript is after all exclusions.<br><br>More information about data exclusions can be found on our OSF page (https://osf.io/g759u/).
Non-participation	52 participants from baseline did not compete the 1 month follow-up, 69 did not complete the 3 moth follow-up, and 94 did not complete the 6 month follow-up. No reasons were provided by participants for not completing these surveys.
Randomization	Participants were not allocated to experimental groups in this study.

March 2021

Reporting for specific materials, systems and methods

We require information from authors about some types of materials, experimental systems and methods used in many studies. Here, indicate whether each material, system or method listed is relevant to your study. If you are not sure if a list item applies to your research, read the appropriate section before selecting a response.

Materials & experimental systems

n/a	Involved in the study
☒	☐ Antibodies
☒	☐ Eukaryotic cell lines
☒	☐ Palaeontology and archaeology
☒	☐ Animals and other organisms
☐	☒ Human research participants
☒	☐ Clinical data
☒	☐ Dual use research of concern

Methods

n/a	Involved in the study
☒	☐ ChIP-seq
☒	☐ Flow cytometry
☒	☐ MRI-based neuroimaging

Human research participants

Policy information about studies involving human research participants

Population characteristics	92.4% students from University of Toronto Scarborough, 63% female, mean age 20.4 years, SD= 5.9
Recruitment	A convenience sample of participants were predominantly recruited through an undergraduate participant pool, though a smaller number were also recruited through on-campus and local advertisements at the University of Toronto Scarborough, for a multi-part study on the effects of physiological and psychological states on goal pursuit . There is the possibility of self-selection bias, particularly participants who wanted the money (up to 75$ CAD), were interested in goal pursuit and/or physiology and psychology, or were willing to invest the time required for participation
Ethics oversight	University of Toronto Research Ethics Board, Social Sciences, Humanities, and Education Committee (Approval number 30380)

Note that full information on the approval of the study protocol must also be provided in the manuscript.
